# In vitro influence of dietary protein and fructooligosaccharides on metabolism of canine fecal microbiota

**DOI:** 10.1186/s12917-016-0672-1

**Published:** 2016-03-12

**Authors:** Carlo Pinna, Carla Giuditta Vecchiato, Giuliano Zaghini, Monica Grandi, Eleonora Nannoni, Claudio Stefanelli, Giacomo Biagi

**Affiliations:** Department of Veterinary Medical Sciences, University of Bologna, via Tolara di Sopra 50, 40064 Ozzano Emilia, Italy; Department for Life Quality Studies, University of Bologna, Corso d’Augusto 237, 47921 Rimini, Italy

**Keywords:** Dog, Dietary protein, Fructooligosaccharides, Intestinal microbiota

## Abstract

**Background:**

The present in vitro study investigated whether the utilization of fructooligosaccharides (FOS) may influence canine fecal microbial population in presence of diets differing in their protein content and digestibility. Fresh fecal samples were collected from five adult dogs, pooled, and incubated for 24 h with the undigested residue of three diets: 1, Low protein high digestibility diet (LP HD, crude protein (CP) 229 g/kg); 2, High protein high digestibility diet (HP HD, CP 304 g/kg); 3, High protein low digestibility diet (HP LD, CP 303 g/kg) that had been previously subjected to enzymatic digestion. In the in vitro fermentation study, there were six treatments: 1) LP HD; 2) HP HD 3) HP LD; 4) LP HD + FOS; 5) HP HD + FOS; 6) HP LD + FOS. Fructooligosaccharides were added at the final concentration of 1.5 g/L. Samples of fermentation fluid were collected at 6 and 24 h of incubation.

**Results:**

Values of pH were reduced by FOS at 6 and 24 h (*P* < 0.001); conversely, low protein digestibility and high dietary protein level resulted in higher pH at both sampling times (*P* < 0.001). At 24 h, FOS lowered ammonia (−10 %; *P* < 0.001) and resulted (*P* < 0.05) in higher concentrations of total volatile fatty acids (VFA) (+43 %), acetic acid (+14 %), propionic acid (+75 %) and *n*-butyric acid (+372 %). Conversely, at 24 h, low protein digestibility resulted (*P* < 0.01) in lower concentrations of acetic acid (−26 %), propionic acid (−37 %) and total VFA (−21 %). Putrescine concentrations were increased at 6 and 24 h of fermentation by low protein digestibility (+21 and 22 %, respectively; *P* < 0.05) and FOS (+18 and 24 %, respectively; *P* < 0.01). After 24 h of fermentation, high dietary protein level resulted in lower counts of lactobacilli and enterococci (−0.5 and −0.7 log cells/mL, respectively; *P* < 0.05) whereas low protein digestibility tended to increase counts of *C. perfringens* (+0.2 log cells/mL; *P* = 0.07).

**Conclusions:**

Results from the present study showed that diets rich in protein may exert negative influences on the canine intestinal ecosystem, slightly increasing the presence of ammonia and reducing counts of lactobacilli and enterococci. Moreover, the presence of poorly digestible protein resulted in lower concentrations of VFA. Conversely, administration of FOS may improve metabolism of canine intestinal microbiota, reducing ammonia concentrations and enhancing VFA production.

## Background

The role of the microbial community that inhabits the large intestine is considered of fundamental importance in the maintenance of gastrointestinal tract health and host physiology. As some studies suggested, major functions of the gut microbiota include protection against potentially pathogenic microorganisms [1], detoxification of some catabolites introduced with the diet [2] and stimulation of the immune system [3]. Exogenous factors, diet above all, can shape the composition and metabolic activity of the intestinal microbiota [4]. Several strategies have been proposed to improve and maintain intestinal microbiota balance, among which the inclusion of prebiotic supplements. Prebiotics (mainly of vegetable origin) are often added to pet food, due to the increasing interest regarding companion animal health and welfare. Despite the fact that dogs are animals with a prevalent carnivorous diet, prebiotic supplements can enhance their gut health. Several positive effects have been seen in dogs receiving prebiotic non-digestible oligosaccharides, including stimulation of beneficial microbes [5], inhibition of undesirable bacteria [6], and reduction of protein-derived catabolites [7, 8]. Moreover, the intestinal microbiota of dogs can be negatively affected by the amount of undigested protein that reaches the colon and leads to putrefactive fermentation [9, 10].

The aim of this study was to assess the in vitro effects of diets differing in their protein content and digestibility, and containing or not a source of fructooligosaccharides (FOS), on the composition and activity of the canine fecal microbiota.

## Methods

### In vitro study

There were three experimental dry extruded diets (provided by Effeffe Pet Food S.p.A., Italy): 1) Low protein high digestibility diet (LP HD; crude protein (CP) 229 g/kg dry matter (DM)); 2) High protein high digestibility diet (HP HD; CP 304 g/kg DM); 3) High protein low digestibility diet (HP LD; CP 303 g/kg DM). All diets were formulated based on the following ingredients: cereals, meat and meat by-products, oils and fat, protein plant extract, minerals and yeasts. The sole source of animal protein in the HD diets was a highly digestible swine meat meal (CP 685 g/kg DM; in vitro digestibility 0.71) whereas the LD diet contained the highly digestible swine meat meal (in the same amount as in the LP HD diet) and a poorly digestible meat meal from cattle and swine (CP 629 g/kg DM; in vitro digestibility 0.31). Digestibility of meat meals was determined using the 2-step procedure proposed by Vervaeke et al. [11] and modified (2 h incubation with HCl, pepsin and gastric lipase followed by 4 h with pancreatin and bile salts) as described by Biagi et al. [12]. The chemical composition of the experimental diets and their undigested residues is shown in Table 1.

**Table 1:** Chemical composition of experimental diets and undigested residues after in vitro digestion

	DM, g/kg	g/kg, DM basis					
		Crude protein	Ether extract	Crude ashes	NDF	ADF	Starch
*Experimental diets*							
LP HD	929	229	120	66	83	27	464
HP HD	935	304	140	82	109	27	364
HP LD	938	303	124	123	111	33	338
*Undigested residues*							
LP HD	-	159	35.7	215	539	175	41.4
HP HD	-	174	47.2	259	481	124	28.7
HP LD	-	212	24.4	339	411	125	9.0

*LP HD* low protein high digestibility, *HP HD* high protein high digestibility, *HP LD* high protein low digestibility, *ADF* acid detergent fiber, *NDF* neutral detergent fiber

The experimental diets were digested using the same 2-step procedure that was used to determine digestibility of meat meals [12]. Digestibility coefficient was 0.84, 0.83 and 0.68 for LP HD, HP HD and HP LD, respectively. After in vitro digestion, the undigested residue of each diet was dried at 65 °C (until a constant dry weight was obtained) and later used in the in vitro fermentation study.

Five healthy adult dogs (household dogs, different breeds, living in different environments, between 1 and 6 years of age, average body weight: 17 kg) were fed for 4 weeks with the LP HD diet added with FOS (from partially hydrolyzed inulin from chicory) at 15 g/kg of food (Effeffe Pet Food Sp.A., Italy). Before the trial started, consent was obtained from each owner. The feeding of a commercial diet to the dogs that were used as fecal donors for the present in vitro study is a procedure that does not fall in the subject matter and scope of the actual legislation on the protection of animals used for scientific purposes. Fresh feces were collected from the five dogs immediately after excretion, pooled and suspended at 10 g/L in pre-reduced Wilkins Chalgren Anaerobe Broth (WCAB). The fecal suspension was used to inoculate (33 mL/L) five 30 ml anaerobic serum bottles (containing 21 ml of medium prepared according to Sunvold et al. [13]) per treatment.

In the in vitro fermentation study, there were 6 treatments: 1) LP HD; 2) HP HD; 3) HP LD; 4) LP HD + FOS; 5) HP HD + FOS; 6) HP LD + FOS. The anaerobic serum bottles contained the undigested residue of the diets, added at 10 g/L. Fructooligosaccharides (Beneo OPS, FOS from partially hydrolyzed inulin from chicory with a dp between 2 and 8; Beneo GmbH, Mannheim, Germany) were added at the final concentration of 1.5 g/L. These concentrations should reflect the amount of FOS that reach the hindgut when they are included in a commercial extruded food for dogs at a concentration of 15 g/kg of food. In fact, if we estimate that the coefficient of digestibility for the dry matter of a commercial super premium dry food for dogs is 0.9, and assuming that all the FOS will reach the large intestine, the ratio between the undigested food fraction and FOS in the hindgut will be approximately 10:1.5.

Fecal cultures pH was adjusted to 6.7; bottles were incubated for 24 h at 39 °C in an anaerobic cabinet (Anaerobic System, Forma Scientific Co., Marietta, USA; under a N_2_ 85 %, CO_2_ 10 %, H_2_ 5 % atmosphere) and samples of fermentation fluid were collected from each bottle at 6 and 24 h and immediately frozen at -80 °C for the determination of ammonia, volatile fatty acids (VFA), biogenic amines and bacterial counts. Additional fresh aliquots were used for pH measurement at 6 and 24 h.

### Chemical and microbiological analyses

Analyses of experimental diets and their undigested residues were performed according to AOAC standard methods [14] (method 950.46 for water, method 954.01 for CP, method 920.39 for ether extract, method 920.40 for starch, method 942.05 for crude ash). Fiber fractions were determined according to the procedure described by Van Soest et al. [15], where neutral detergent fiber (NDF) was assayed with a heat stable amylase and expressed inclusive of residual ash, acid detergent fiber (ADF) was expressed inclusive of residual ash.

Ammonia was measured using a commercial kit (Urea/BUN – Color, BioSystems S.A., Barcelona, Spain). Volatile fatty acids were analyzed by gas chromatography [16]. For the determination of biogenic amines, samples were diluted 1:5 with perchloric acid (0.3 M); biogenic amines were later separated by high performance liquid chromatography and quantified through fluorimetry, according to the method proposed by Stefanelli et al. [17].

The Fluorescence In Situ Hybridization technique was used to determine bacterial counts. For this purpose, ready-to-use commercial kits (BioVisible B.V., Groeningen, Netherlands) containing specific FITC-labeled probes for the enumeration of enterococci (*E. faecalis* + *E. faecium*), Enterobacteriaceae, *Clostridium perfringens*, *Bifidobacterium* spp. and *Lactobacillus* spp. were used. The slides were evaluated with a Nikon Eclipse E-600 epifluorescence microscope, equipped with FITC specific filter.

### Statistical analyses

Data were analyzed by three-way ANOVA, with dietary protein content and digestibility and FOS as the main effects. Differences were considered statistically significant at *P* < 0.05. All the statistical computations were performed with Statistica 10.0 (Stat Soft Italia, Italy). Due to the fact that the experimental design did not comprise a low protein low digestibility (LP LD) diet, interactions among main effects were not reported. The reason for the absence of the LP LD diet is that the experimental diets used in the present in vitro study were also used in an in vivo study with dogs, where the feeding of a low protein low digestibility diet would have been unacceptable for ethical reasons.

## Results

The pH values at 6 and 24 h of incubation are shown in Table 2. After 6 h of incubation, pH was influenced (*P* < 0.001) both by protein level (6.26 vs. 6.61 for LP and HP, respectively) and digestibility (6.38 vs. 6.71 for HD and LD, respectively), and reduced by FOS supplementation (6.23 vs. 6.75). At 24 h, pH values were lowered (*P* < 0.001) by high protein digestibility (5.98 vs. 6.30), low dietary protein level (6.09 vs. 6.40) and FOS (5.96 vs. 6.44).

**Table 2:** pH values and ammonia concentrations (mmol/L) after 6 and 24 h of the vitro study^a^

							ANOVA *P*-value			
	LP HD	HP HD	HP LD	LP HD + FOS	HP HD + FOS	HP LD + FOS	Protein level	Protein digestibility	FOS	Pooled SEM
At 6 h										
pH	6.60	6.76	6.90	5.93	6.24	6.52	<0.001	<0.001	<0.001	0.04
NH_3_	37.3	37.8	36.0	33.6	35.2	35.1	0.238	0.281	0.002	0.89
At 24 h										
pH	6.23	6.41	6.67	5.74	5.99	6.14	<0.001	<0.001	<0.001	0.03
NH_3_	40.3	41.6	39.2	36.0	37.8	35.6	0.023	0.002	<0.001	0.64

*LP HD* low protein high digestibility, *HP HD* high protein high digestibility, *HP LD* high protein low digestibility, *FOS* fructooligosaccharides

^a^Values are the means of five bottles per treatment

The concentrations of ammonia are shown in Table 2. At 6 h of incubation, ammonia concentration was reduced by the presence of FOS (34.6 vs. 37.0 mmol/L; *P* < 0.01). After 24 h, ammonia was reduced by FOS (36.4 vs. 40.3 mmol/L; *P* < 0.001) and influenced by protein digestibility (38.9 vs. 37.4 mmol/L for HD and LD, respectively; *P* < 0.01) and protein level (38.5 vs. 38.1 mmol/L for HP and LP, respectively; *P* < 0.05).

Concentrations of VFA are shown in Table 3. After 6 h of fermentation, FOS reduced concentrations of propionic and iso-butyric acid (0.73 vs. 1.45 mmol/L and 0.05 vs. 0.15 mmol/L, respectively; *P* < 0.05). The high dietary protein level resulted in lower concentrations of *n*-butyric acid (2.21 vs. 5.14 mmol/L; *P* < 0.05) whereas low protein digestibility determined lower concentrations of acetic acid and total VFA (9.1 vs. 15.3 mmol/L and 11.6 vs. 20.7 mmol/L, respectively; *P* < 0.05). The acetic to propionic acid ratio and acetic acid + *n*-butyric acid to propionic acid ratio were increased (*P* < 0.05) by FOS. After 24 h of fermentation, FOS resulted (*P* < 0.001) in higher concentrations of total VFA (47.1 vs. 32.9 mmol/L; *P* < 0.001), acetic acid (27.2 vs. 23.9 mmol/L; *P* < 0.05), propionic acid (9.93 vs. 5.69 mmol/L; *P* < 0.05), and *n*-butyric acid (9.53 vs. 2.56 mmol/L; *P* < 0.001). Both the acetic to propionic acid ratio and acetic acid + n-butyric acid to propionic acid ratio were reduced by FOS and increased by low protein digestibility (*P* < 0.01). Low protein digestibility also resulted (*P* < 0.01) in lower concentrations of total VFA (33.9 vs. 43.1 mmol/L), acetic acid (20.7 vs. 28.0 mmol/L) and propionic acid (5.63 vs. 8.89 mmol/L).

**Table 3:** Concentrations (mmol/L) of volatile fatty acids at 6 and 24 h of the vitro study^a^

							ANOVA *P*-value			
	LP HD	HP HD	HP LD	LP HD + FOS	HP HD + FOS	HP LD + FOS	Protein level	Protein digestibility	FOS	Pooled SEM
6 h										
Acetic acid	15.2	17.6	9.7	16.8	11.2	8.8	0.304	0.005	0.220	1.44
Propionic acid	1.58	1.56	1.09	1.04	0.59	0.52	0.236	0.214	<0.001	0.18
iso-Butyric acid	0.16	0.15	0.12	0.10	0.02	0.03	0.246	0.731	0.011	0.04
*n*-Butyric acid	2.81	3.10	1.04	7.47	2.33	2.12	0.040	0.304	0.052	1.04
iso-Valeric acid	0.03	0.01	0.04	0.01	0.01	0.01	0.207	0.304	0.135	0.01
Total VFA	19.8	22.4	12.0	25.5	14.2	11.4	0.090	0.019	0.809	2.30
C2:C3	10.3	11.8	9.1	16.4	23.8	26.9	0.457	0.879	0.030	5.53
C2 + *n-*C4:C3	12.3	13.7	10.0	23.6	28.1	30.3	0.616	0.997	0.005	5.43
24 h										
Acetic acid	27.4	24.2	18.8	28.1	33.2	21.8	0.862	0.003	0.037	2.23
Propionic acid	7.65	6.37	1.93	12.12	10.88	7.86	0.105	<0.001	<0.001	0.66
iso-Butyric acid	0.09	0.52	0.16	0.27	0.09	0.27	0.201	0.500	0.483	0.11
*n*-Butyric acid	2.12	3.79	1.11	9.72	7.97	10.67	0.880	0.848	<0.001	0.90
iso-Valeric acid	0.44	0.68	0.23	0.44	0.11	0.22	0.950	0.303	0.106	0.14
Total VFA	37.7	35.5	22.3	50.6	52.3	40.8	0.864	0.001	<0.001	2.66
C2:C3	3.65	3.81	9.61	2.35	3.07	2.77	0.585	0.006	<0.001	0.70
C2 + *n-*C4:C3	3.91	4.40	10.22	3.14	3.86	4.13	0.409	0.001	0.001	0.65

*LP HD* low protein high digestibility, *HP HD* high protein high digestibility, *HP LD* high protein low digestibility, *FOS* fructooligosaccharides, *VFA* volatile fatty acids, *C2/C3* acetic acid/propionic acid ratio, *C2 + n-C4/C3* acetic acid + n-butyric acid/propionic acid ratio

^a^Values are the means of five bottles per treatment

With regard to biogenic amines (Table 4), spermine concentrations after 6 h of incubation were affected by protein level (39.9 vs. 32.3 μmol/mL for LP and HP, respectively; *P* < 0.001). Putrescine concentrations were increased at 6 and 24 h of fermentation by low protein digestibility (+21 and +22 %, respectively; *P* < 0.05) and FOS (+18 and +24 %, respectively; *P* < 0.01). At 24 h, low protein digestibility resulted in higher spermidine (97.8 *vs* 71.4 μmol/mL; *P* < 0.001), whereas FOS induced an increase in spermine concentration (19.8 vs. 9.7 μmol/mL; *P* < 0.001). Cadaverine concentrations were not affected by treatments.

**Table 4:** Concentrations of biogenic amines (μmol/L) at 6 and 24 h of the in vitro study^a^

							ANOVA *P*-value			
	LP HD	HP HD	HP LD	LP HD + FOS	HP HD + FOS	HP LD + FOS	Protein level	Protein digestibility	FOS	Pooled SEM
At 6 h										
Putrescine	443	542	601	591	577	705	0.241	0.013	0.003	35.2
Cadaverine	18.6	25.8	19.0	23.3	12.9	22.5	0.413	0.631	0.133	4.62
Spermidine	63.2	68.8	65.0	68.3	66.4	59.4	0.839	0.519	0.133	6.56
Spermine	40.3	34.5	35.6	41.2	28.6	29.0	<0.001	0.422	0.080	2.08
At 24 h										
Putrescine	550	617	790	780	775	863	0.514	0.013	<0.001	50.0
Cadaverine	31.1	20.9	20.3	24.2	24.5	22.3	0.734	0.220	0.720	6.40
Spermidine	105.4	65.6	67.5	94.0	82.6	66.0	0.002	0.128	0.769	6.18
Spermine	11.9	5.8	11.6	25.8	20.6	17.1	0.055	0.519	<0.001	2.14

*LP HD* low protein high digestibility, *HP HD* high protein high digestibility, *HP LD* high protein low digestibility, *FOS* fructooligosaccharides

^a^Values are the means of five bottles per treatment

Bacterial counts after 6 and 24 h of incubation are shown in Table 5. At 6 h, high dietary protein level resulted in lower counts of *Clostridium perfringens* (5.90 vs. 6.71 log cells/mL; *P* < 0.05), *Lactobacillus* spp. (3.46 vs. 4.42 log cells/mL; *P* < 0.001) and enterococci (7.71 vs. 8.52 log cells/mL; *P* = 0.026). After 24 h of fermentation, high dietary protein level resulted in lower counts of *Lactobacillus* spp. (3.2 vs. 3.7 log cells/mL, *P* < 0.05) and enterococci (7.5 vs. 8.2 log cells/mL; *P* < 0.05), low protein digestibility tended to increase counts of *C. perfringens* (6.0 vs. 5.8 log cells/mL; *P* = 0.07) and FOS resulted in higher Enterobacteriaceae (8.6 vs. 8.2 log cells/mL; *P* < 0.001) and lower *Lactobacillus* spp. (3.1 vs. 3.6 log cells/mL; *P* < 0.001). Bifidobacteria were not affected by treatments (data not shown) and averaged 6.72 and 6.85 log cells/mL at 6 and 24 h of incubation, respectively.

**Table 5:** Bacterial counts (log cells/mL) at 6 and 24 h of the in vitro study^a^

							ANOVA *P*-value			
	LP HD	HP HD	HP LD	LP HD + FOS	HP HD + FOS	HP LD + FOS	Protein level	Protein digestibility	FOS	Pooled SEM
At 6 h										
Enterobacteriaceae	8.60	7.59	8.60	8.70	8.90	8.81	0.404	0.345	0.177	0.47
*C. perfringens*	6.74	5.20	6.59	6.68	5.93	5.89	0.049	0.231	0.976	0.55
*Lactobacillus* spp.	4.31	3.60	3.41	4.53	3.45	3.37	<0.001	0.303	0.929	0.13
Enterococci	8.54	7.20	7.80	8.50	7.81	8.13	0.026	0.297	0.405	0.43
At 24 h										
Enterobacteriaceae	8.31	8.43	7.94	8.69	8.51	8.69	0.746	0.097	<0.001	0.09
*C. perfringens*	5.83	5.79	5.98	5.81	5.88	6.08	0.918	0.068	0.499	0.10
*Lactobacillus* spp.	4.20	3.34	3.16	3.16	3.00	3.12	0.002	0.836	<0.001	0.14
Enterococci	8.04	7.86	7.69	8.30	6.72	7.66	0.040	0.349	0.371	0.41

*LP HD* low protein high digestibility, *HP HD* high protein high digestibility, *HP LD* high protein low digestibility, *FOS* fructooligosaccharides

^a^Values are the means of five bottles per treatment

## Discussion

Due to ethical and economic concerns regarding the use of animals for scientific purposes, in vitro fermentation models have been widely used to investigate the effects of dietary factors on gut microbiota, both in humans and animals [13, 18–22]. The aim of the present study was to investigate whether FOS may influence fecal microbial population of dogs in presence of diets differing in their protein content and digestibility.

The reduction of colonic luminal pH has potential positive effects on the host gut health, due to the inhibiting effect that an acidic environment has on some harmful bacteria [23]; moreover, a low pH can induce a shift from ammonia to ammonium ions, thus limiting the absorption of ammonia across the intestinal mucosa [24]. In the present study, pH was reduced by FOS whereas high dietary protein level and low protein digestibility resulted in higher pH values. The higher pH values that were found in bottles containing the high dietary protein level may be the consequence of the slightly higher ammonia concentrations that were observed at 24 h. On the other hand, at 6 and 24 h, low protein digestibility resulted in lower VFA concentrations and this finding may explain the higher pH values observed in the vessels containing the LD diets. It is known that bacterial fermentation of carbohydrates leads to the production of VFA and lactic acid which, in turn, lower the intestinal pH. In fact, in the present study, FOS resulted in higher concentrations of VFA. In a previous in vitro experiment with canine fecal inoculum, FOS fermentation resulted in lower pH values than control [12]. Conversely, in another in vitro study, FOS did not affect pH when fermented in presence of feline fecal inoculum [25]. Moreover, in several in vivo studies with dogs, dietary supplementation with FOS failed to affect fecal pH [26–29]. However, it is well known that the concentration of VFA can vary while digesta move along the intestine as these metabolites are absorbed by the intestinal mucosa [30]. According to Topping and Clifton [31], only 5 % of total VFA produced in the intestine by bacterial fermentation may be recovered in the feces. This could explain the discrepancy in pH values and VFA concentrations often found between in vitro and in vivo studies.

Fermentation of experimental diets resulted in different VFA concentrations. After 24 h of fermentation, the presence of FOS resulted in higher concentrations of acetic, propionic and *n*-butyric acids. In particular, higher intestinal concentrations of *n*-butyric acid may improve the animal’s intestinal health because this acid is the main energy source for the epithelial cells of the terminal ileum [32] and hindgut [33]. In agreement with our findings, other authors reported higher fecal concentrations of total VFA [34], *n*-butyric acid [26, 35] and propionic acid, the latter under both in vitro [12] and in vivo [26, 27] conditions, when FOS were added to canine diets. With regard to dietary protein, VFA production was reduced by low protein digestibility but was not influenced by dietary protein level. This result suggests that microbial fermentations in the canine hindgut may be affected more by protein digestibility than by protein content of the diet. However, it also has to be noticed that LD diets contained slightly less starch than the HD diets, which may partially explain the different VFA concentrations that were observed. In a recent study with dogs, feeding a high-protein greaves-meal diet (609 g of CP per kg of diet) resulted in lower acetic and propionic acids and higher branched-chain fatty acids (BCFA) fecal concentrations than control (control diet contained 264 g of CP per kg of diet), but digestibility of experimental diets was not evaluated [36]. In the present study, LD diets reduced concentrations of both acetic and propionic acids whereas HP diets had no significant effect on the concentrations of these microbial metabolites.

As already mentioned, high dietary protein level resulted in higher ammonia concentrations. Higher fecal ammonia concentrations in dogs fed protein-rich diets were reported by other authors [9, 28, 36, 37]. In a recent study, Nery et al. [10] reported greater fecal ammonia concentrations in dogs fed diets containing poultry meal if compared with highly digestible wheat gluten diets; in the same study, fecal ammonia concentrations were increased when dogs were fed diets containing high levels of protein (CP 390 g/kg of diet) if compared with low-protein diets (CP 220 g/kg of diet). In another study, Hesta et al. [28] noticed increased fecal ammonia concentrations in dogs fed diets supplemented with meat and bone meal or greaves meal but not when diets were supplemented with poultry meal. Interestingly, in the present study, low protein digestibility did not increase ammonia concentrations. This result is difficult to explain considering the fact that the undigested residue of the LD diet contained more protein (212 g/kg of CP) than the undigested fraction of the HD diets (159 and 174 g/kg of CP for LP and HP, respectively). Ammonia is a toxic compound that can be the cause of reduced villus height [38] and even have carcinogenic effects [39]. In humans [40, 41] and dogs [7], the treatment of renal and hepatic failure involves reducing the circulating ammonia by using prebiotics or antibiotics, thus reducing ammonia production by intestinal bacteria. In fact, prebiotic ingredients represent a source of energy for saccharolytic bacteria [42, 43] and contribute to restricting proteolytic fermentation and promoting nitrogen utilization by colonic bacteria [7]. In the present study, ammonia concentrations were reduced by FOS both after 6 and 24 h of incubation. Conversely, in previous in vitro studies with canine [12] and feline [25] fecal inocula, the incubation of FOS failed to reduce ammonia concentrations. Inconsistent results have been obtained also under in vivo conditions when dogs were fed diets containing FOS. In fact, while Flickinger et al. [27] observed that FOS tended to reduce fecal ammonia concentrations in dogs, other authors [8, 26, 28, 44, 45] failed to observe any positive influence of FOS on canine fecal ammonia. It seems evident that the effect of FOS on intestinal and fecal ammonia concentrations in dogs can be influenced by several variables, including the amount of FOS used, as well as dietary and environmental factors. Moreover, like VFA, ammonia is easily absorbed through the intestinal mucosa and its fecal concentration might not be representative of the gut concentration.

While VFA are derived from both protein and carbohydrate fermentation, BCFA derive exclusively from branched amino acids bacterial breakdown [46–48]. It has been shown that metabolites deriving from bacterial fermentation of protein in the gut are affected both by dietary protein content and source in humans [49] as well as in dogs [10, 36, 50] and other carnivorous animals, such as cheetahs [21]. However, in the present study, iso-butyric and iso-valeric acids concentrations were not affected by protein level and digestibility. Conversely, at 6 h of fermentation, the presence of FOS resulted in a reduction of iso-butyric acid concentration. This finding is in accordance with Depauw et al. [21] who reported a reduction of total BCFA when cheetah fecal inoculum was incubated in presence of FOS.

Biogenic amines are putrefactive compounds derived from amino acid and peptide decarboxylation [51]. Biogenic amines such as histamine and tyramine play important roles in maintaining the physiology of animals but also have toxicological properties, therefore constituting a potential health risk [52]. Moreover, other biogenic amines such as cadaverine, putrescine, spermidine and spermine do not seem to have direct toxic effects but can potentiate histamine and tyramine toxicity by competing with detoxifying enzymes [53]. In the present study, fermentation of FOS resulted in increased concentrations of putrescine and spermine. This finding is in apparent contradiction with the reduction of other proteolytic compounds, such as ammonia and iso-butyric acid, that was observed in vessels containing FOS. Recently, in an in vitro study with feline fecal inoculum, fermentation of FOS, galacto-oligosaccharides and pectin resulted in increased concentrations of putrescine [25]. Similarly, Barry et al. [54] observed increased fecal concentrations of cadaverine and putrescine in cats receiving FOS or pectins. Beloshapka et al. [55] noticed an increase of fecal spermine in dogs receiving two different raw beef and chicken-based diets added with inulin and yeast cell wall. Moreover, Propst et al. [35] found higher fecal concentrations of biogenic amines in dogs fed increasing levels of FOS. Conversely, in the study by Flickinger et al. [27], feeding dogs with increasing levels of FOS did not affect fecal concentrations of putrescine and spermidine, while cadaverine and spermine were reduced. Spano et al. [56] observed that lactic acid bacteria (LAB) strains may produce biogenic amines by enzymatic decarboxylation of amino acids when exposed to acidic stress conditions. Therefore, since the fermentation of FOS resulted in lower pH values, the higher concentrations of cadaverine and putrescine that were observed in the present study may be the result of acid tolerance mechanisms activated by LAB. On the other hand, low protein digestibility resulted in higher concentrations of putrescine, presumably because of increased proteolysis. Interestingly, concentrations of spermine at 6 h and spermidine at 24 h of fermentation were lowered by high dietary protein level. At present, we do not have an explanation for this finding. However, based on the present results, biogenic amines, unlike ammonia and BCFA, do not seem to represent an accurate indicator for bacterial proteolytic metabolism.

After 24 h of incubation, high dietary protein level resulted in lower counts of lactobacilli and enterococci whereas low protein digestibility tended to increase counts of *C. perfringens*. Similar results were observed in other studies with dogs [9, 37] where the administration of diets rich in animal derived protein resulted in increased growth of proteolytic bacteria at the expense of LAB, and these effects were even more evident when dogs were fed diets based on low quality protein sources. Conversely, Nery et al. [10] did not observe any variation in *C. perfringens* fecal counts in dogs receiving diets formulated with different protein content and protein sources. According to the definition given by Roberfroid [57], prebiotics are non-digestible substances that are added to an animal’s diet to provide a source of energy for beneficial bacterial strains residing in the hindgut. As such, prebiotics would be expected to increase intestinal counts of beneficial bacteria, such as LAB and bifidobacteria. In the present study, FOS failed to increase fecal counts of LAB and bifidobacteria and even resulted, after 24 h of incubation, in lower counts of *Lactobacillus* spp. Several studies with sometimes conflicting results have been conducted in order to investigate the effects of FOS administration on the composition of canine intestinal microbiota. In a study by Swanson et al. [26], feeding adult dogs with 2 g of FOS per day resulted in higher counts of bifidobacteria and lactobacilli. Conversely, Flickinger et al. [27] found that the use of FOS reduced *C. perfringens* in dog feces, without exerting any influence on the fecal population of lactobacilli and bifidobacteria. Similarly, other authors [8, 44, 58] reported no effects of FOS administration on fecal counts of LAB and bifidobacteria in dogs. It has been reported by several authors [59–61] that bifidobacteria are inconsistently detected in canine feces. However, other authors [26, 29] found greater fecal concentrations of bifidobacteria in dogs. In the present study, bifidobacteria were not affected by experimental diets and their concentration in fermentation liquid was 6.8 log cells/mL. However, in this study, feces from dogs used as donors were not individually analyzed so that is not possible to know if bifidobacteria were present in all animals. Finally, it is worthy to mention that bacterial populations were analyzed by DNA analysis techniques in only a few of the cited studies [8, 29, 37, 60, 61], while in the others bacteria were enumerated on selective media [9, 10, 26, 27, 44, 58, 59].

## Conclusions

Results from the present study showed that diets rich in protein may exert negative influences on the canine intestinal ecosystem, slightly increasing the presence of ammonia and reducing counts of lactobacilli and enterococci. Moreover, the presence of poorly digestible protein resulted in lower concentrations of VFA. Conversely, administration of FOS may improve metabolism of canine intestinal microbiota, reducing ammonia concentrations and enhancing VFA production.

## Abbreviations

ADF: acid detergent fiber
BCFA: branched-chain fatty acids
CP: crude protein
DM: dry matter
dp: degree of polymerization
FOS: fructooligosaccharides
HP HD: high protein high digestibility diet
HP LD: high protein low digestibility diet
LAB: lactic acid bacteria
LP HD: low protein high digestibility diet
LP LD: low protein low digestibility diet
NDF: neutral detergent fiber
VFA: volatile fatty acids
WCAB: Wilkins Chalgren Anaerobe Broth
